# Nonselective Beta-Blockers for the Efficacious Healing of Ulcerated Infantile Hemangiomas in Unusual Locations of Two Female Infants

**DOI:** 10.7759/cureus.16683

**Published:** 2021-07-28

**Authors:** Divya Poulose, Samruddhi Lote, Aditi Mahajan, Jaya Madhurya Gogineni

**Affiliations:** 1 Dermatology, Dr. D.Y. Patil Medical College Hospital and Research Centre, Pune, IND; 2 Dermatology, Dr. D.Y. Patil Medical College, Hospital and Research Centre, Pune, IND

**Keywords:** infantile hemangioma, propranolol, timolol, soft tissue tumors, ulceration

## Abstract

Infantile hemangiomas (IHs) are the most common vascular soft-tissue tumors of newborns and early childhood with a prevalence rate of approximately 4%-5% in infancy. Most of the IHs regress spontaneously after initial proliferation. Ulceration is one of the most common complications seen in IHs, which heal poorly without treatment. The prevalence of IHs is three times more in females compared to males. The most common location of IH is head and neck with the least being in extremities Oral propranolol and systemic corticosteroids are used as first-line therapy in the treatment. Topical timolol has been used as an alternative to counteract the long-term side effects of the prior modalities. In this study, we are reporting and presenting case reports of two female infants with ulcerated IH who were treated with oral propranolol and topical timolol, respectively, and comparing the efficacy of one over the other. we observed fast and efficacious healing with oral propranolol when used with proper monitoring, as compared to topical timolol, although the latter is more commonly used as it is safer with fewer potential side effects.

## Introduction

Infantile hemangiomas are the most frequently seen vascular soft-tissue tumors of newborns and pediatric age groups. Most infantile hemangiomas regress spontaneously after initial growth and no treatment is needed unless there are complications. The most frequent complication is ulceration, which is associated with pain, bleeding, infection, and scarring and heals poorly without treatment [1]. Other infantile hemangioma-related complications are high-output cardiac failure, functional and postural difficulties, airway compromise, ophthalmologic problems like astigmatism, and permanent disfigurement [2]. The prevalence rate is approximately 4%-5% in infants [3]. Infantile hemangiomas are due to multiple antenatal risk factors like vaginal bleeding, use of progesterone, toxemia of pregnancy, late pregnancy, and low-lying placenta [4]. Family history was also identified as a risk factor for infantile hemangioma [5]. The first line of therapy is systemic nonselective beta-blockers (oral propranolol) and corticosteroids, but topical timolol maleate has also been used as a safe, new, off-label treatment modality [6]. Both the cases of ulcerated infantile hemangioma reported here were seen in uncommon locations.

## Case presentation

Two female infants aged seven months (child X) and nine months (child Y), presented to us, with complaints of swelling in the right axilla and below the right gluteal region, respectively. Parents of both the children gave the history of no such swelling at the time of birth and were seen at five weeks and nine weeks after birth in child X and child Y, respectively. The growth and development of both the children were not affected and the milestones were achieved normally. On examination, it was noted that swelling of size 4*5 cm, reddish-brown in color with central ulceration was seen in child X on the left axilla, whereas child Y was presented with pale red swelling of size 7*3 cm with erythema in some areas and central ulceration located below the right gluteal region.

**Figure 1:**
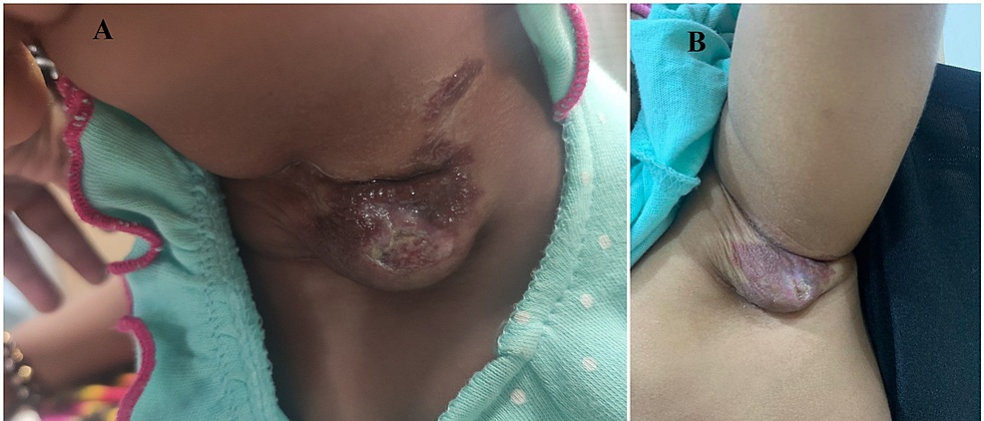
child X : A) Ulcerated infantile hemangioma in the left axilla of size 4*5 cm with central ulceration, reddish brown in color before treatment with topical timolol. B) After five weeks of treatment with topical timolol 0.5%

**Figure 2:**
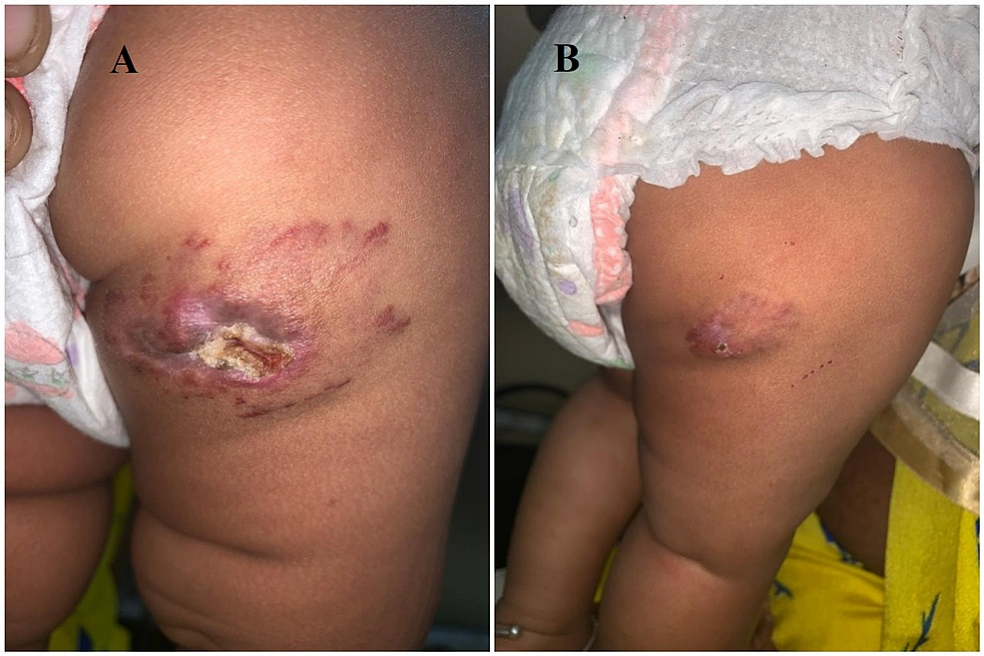
Child Y: A) Ulcerated hemangioma of size 7*3 cm, red in color with central ulceration below the right gluteal region before oral propranolol. B) After four weeks of therapy with oral propranolol 1 mg/kg

After a complete evaluation and cardiac workup, both the children were admitted, child X was treated with local application of topical timolol 0.5%, three drops twice daily, and child Y with oral propranolol 1 mg/kg divided into three equal doses daily. The pulse, heart rate, respiratory rate, and blood pressure were monitored every 30 minutes, for 4 hours after each dose for both the children. A significant reduction in the size, color, and texture of the hemangiomas was seen with oral propranolol within four weeks (Figure 1), and topical timolol within five weeks (Figure 2), with complete healing seen within nine weeks and 15 weeks, respectively. There have been no local and systemic adverse effects, with no recurrence of ulcers in both cases since the last six months.

## Discussion

Infantile hemangiomas can be grouped into superficial, deep, and mixed, which may present as a solitary lesion or multiple, and can range from a few millimeters to centimeters in size [7]. Superficial lesions appear to be firm in consistency, raised, red papules, nodules, or plaques, whereas the lesions that extend deep down appear to be skin-colored with a bluish tint. Mixed lesions show characteristic features of both [2]. Deep hemangiomas tend to grow rapidly in the late phase than superficial hemangiomas, and they progress for a longer period of up to two years [5]. Infantile hemangiomas can also be classified as localized or segmental. Localized lesions are seen as nodules or plaques in a limited area, but segmental lesions take the appearance of linear or geometric arrangements related to the area of developmental growth [2]. The pathogenesis is not well understood and various theories have been proposed, such as a response to hypoxia, somatic mutation, angiogenic peptides, placental embolization, and proliferation of progenitor cells that travel to hypoxic areas in the infants. The diagnosis of hemangiomas is based on history, physical examination, diagnostic tools such as magnetic resonance imaging and color Doppler ultrasonography [8]. Skin Lesion biopsy has to be done for hemangiomas with atypical appearance and features with unclear etiology [9]. Numerous treatment modalities have been used to treat infantile hemangiomas. Propranolol is effective in stopping the growth during the proliferative phase, also quickening the involution phase and it also leads to early apoptosis of endothelial cells, thereby reducing the size of the tumor. The side effects of propranolol are bradycardia, hypotension, hypoglycemia, gastrointestinal discomfort/reflux, fatigue, and bronchospasm. Vincristine and interferon-alpha have been used to treat aggressive infantile hemangiomas that do not respond to systemic corticosteroids [1,10]. In a study done by Hogeling et al. [11], propranolol was given at 2 mg/kg, which led to a reduction in size, change in color up to the age of five years. Topical timolol was found to be successful in the treatment of early lesions with the advantages of being low cost, easy administration, and minimal adverse effects [12]. Systemic glucocorticoids have been used to stop tumor growth in the early proliferative phase, but are less useful at later stages. Long-term side effects of systemic glucocorticoids are skin depigmentation, atrophy of skin and fat, and necrosis [13]. In our study, there was a significant reduction in the size, color, and texture of the hemangiomas with topical timolol within four weeks and oral propranolol within five weeks, with complete healing seen within 15 weeks and nine weeks of child X and Y, respectively. We have documented no local and systemic adverse effects, with no recurrence of ulcers in both cases since the last six months.

## Conclusions

Beta-blockers have revolutionized the treatment of infantile hemangiomas. Physicians and specialists should be cautious in managing infantile hemangioma, keeping in mind the complications of the tumor and adverse effects of treatment modalities. Doctors are recommended to monitor the infants with regular follow-up and counseling the parents to look out for any after-effects. From our study, we noticed that both the cases of ulcerated infantile hemangiomas were seen in uncommon locations with fast and efficacious healing with oral propranolol when used with proper monitoring, as compared to topical timolol, although the latter is more commonly used as it is safer with fewer potential side effects.
